# Parental drinking according to parental composition and adolescent binge drinking: findings from a nationwide high school survey in Japan

**DOI:** 10.1186/s12889-020-09969-8

**Published:** 2020-12-07

**Authors:** Satoshi Inoura, Takuya Shimane, Kunihiko Kitagaki, Kiyoshi Wada, Toshihiko Matsumoto

**Affiliations:** 1 Department of Drug Dependence Research, National Institute of Mental Health, National Center of Neurology and Psychiatry, Tokyo, Japan; 2 Social Pharmacy Lab., Faculty of Pharmaceutical Sciences, Tokyo University of Pharmacy and Life Sciences, Tokyo, Japan; 3 Department of Addiction Treatment Research, Saitama Prefectural Psychiatric Hospital, Saitama, Japan

**Keywords:** Parental drinking, Parental composition, Binge drinking, Adolescent, High school, School survey

## Abstract

**Background:**

Alcohol problems in parents have been revealed to affect adolescent alcohol misuse. However, few studies examine the effects of parental drinking on adolescent risky drinking (including binge drinking) in the general population. In particular, previous study findings are inconsistent regarding the influence of parental drinking according to parental composition. In this study, we aimed to examine the relationship between parental drinking, according to parental composition, and binge drinking among high school students in Japan.

**Methods:**

We performed a secondary analysis of the Nationwide High School Survey on Drug Use and Lifestyle 2018, Japan. A total of 46,848 valid surveys from high school students of 78 schools were included for analysis. Logistic regression analysis with a generalized linear mixed model was conducted with binge drinking as the dependent variable and “parental drinking according to parental composition” (e.g., father’s drinking, mother’s drinking, father’s absence, mother’s absence, both parents drinking, and neither parent at home) as the independent variable, after adjusting with covariates. Binge drinking was defined as five or more alcoholic drinks for male adolescents or four or more alcoholic drinks for females on the same occasion within two hours.

**Results:**

In the fully adjusted models, adolescents whose mothers drink (adjusted odds ratio (AOR): 1.50, 95% confidence interval (CI): 1.06–2.12) were significantly associated with adolescent binge drinking. This risk was significantly higher among students with neither parent living at home (AOR: 4.35, 95% CI: 2.10–9.02).

**Conclusion:**

Parental drinking and absence do affect adolescent binge drinking; our findings show that adolescents are more likely to engage in binge drinking if their mothers drink or if they are not living with either parent. Therefore, it is important to engage parents and non-parental family members in future programs and interventions to prevent adolescent binge drinking.

## Background

Alcohol misuse is common among adolescents. A recent survey found that approximately 2.4% of high school students in Japan [1] engage in binge drinking, which is lower than the data from other countries, including the United States (14.4% of 12th graders [2]) and Europe (35.0% of adolescents aged 16 years [3]). Although the prevalence of binge drinking in Japan is low when compared internationally, we should not make light of this problem because youth binge drinking increases the risk of alcohol-related disorders (including intoxication), accidents, fatal injuries, and chronic illness [4]. Binge drinking also increases the risk of both acute and long-term negative consequences (e.g., injuries and alcohol disorders) [5], essentially, effecting further risk behaviors such as illegal substance use and sexual and violent behaviors [6–8]. Japanese culture includes a deep-rooted phenomenon similar to alcohol chugging. Often observed in society and media advertisements [9], it relates to risky heavy drinking, promoting positive perceptions and attitudes about alcohol consumption, and potentially affecting children’s beliefs and behaviors toward alcohol consumption. In fact, heavy underage and college drinking have been reported recently [10, 11], making it essential to raise awareness of the dangers of binge drinking and understanding and addressing the environmental factors surrounding adolescents, including the person themselves, at an early stage.

Emerging health research suggests that alcohol use is determined by multidimensional (including biological, psychological, and sociocultural) factors [12]. Genetic variants affect the risk of alcohol misuse. Candidate genes related to alcohol dependence can be categorized into three groups: those related to (a) alcohol metabolism, (b) the stress response system, and (c) behavioral disinhibition [13, 14]. With respect to alcohol metabolism, alcohol dehydrogenase and aldehyde dehydrogenase are known to have a protective influence against excessive drinking and alcohol dependence [15]. Regarding the stress response system, the initiation and retention of binge drinking behavior and alcohol dependence are related through dysregulated activation of the brain stress system by corticotropin-releasing hormones [16, 17]. Behavioral disinhibition, defined as the inability to inhibit socially restricted actions, including alcohol and other substance abuse, is related to the serotonin and dopamine pathways [18, 19]. Previous studies have associated polymorphisms of tryptophan hydroxylase, serotonin transporter and serotonin receptor genes in the serotonergic system, polymorphisms on dopamine receptors, dopamine β-hydroxylase, and tyrosine hydroxylase with risks of binge drinking and alcohol dependence [14, 20–22].

Psychological traits related to cognitive and emotional susceptibility to substance abuse are also crucial factors for the initiation and development of adolescent substance abuse [23]. Among these, impulsivity and sensation seeking have received the most attention owing to their influence on risky behaviors (including drinking alcohol) [24, 25] with high levels being especially influential [26]. Adolescents, in particular, may be more sensitive to alcohol’s reward and stimulant effects [27], but less so to its sedative effects [28]. Several studies imply that neural changes during adolescence may temporarily increase sensitivity to certain effects (e.g., reward) of alcohol, promoting consumption during a drinking episode. It may also decrease sensitivity to other effects (e.g., sedative effects) that may help to limit drinking during an episode [29].

Regarding sociocultural factors, etiological mechanisms representing multiple systems (including family, peers, and community) interact across development to influence binge drinking tendencies [30]. Here, family plays an essential role. Not only does parental drinking have a crucial influence on children, but studies [31, 32] show that disrupted family relationships (including parental separation or divorce) and parental intactness are associated with binge drinking. Furthermore, parental control and support are essential in preventing adolescents from engaging in risky drinking behaviors [33], since low warmth and low parental monitoring regarding their child’s alcohol use are associated with higher adolescent binge drinking prevalence [34]. Importantly, binge drinking occurs where there is no parental supervision, such as someone else’s home [35]. During high school, an adolescent’s social context shifts from family to peers. Binge drinking may be facilitated by the presence of peers and peer selection [36, 37]. Community factors include the neighborhood and school environment. A supportive school environment (e.g., incorporating alcohol prevention into the curriculum) is associated with reduced adolescent binge drinking independent of individual, family, and peer risk factors [38].

Recent studies have focused on the influence of alcohol use on others, including the potential impact of parental drinking on children, especially adolescent alcohol misuse. Whereas most studies focused on alcoholics and parents who had problems with alcohol [39], some recent ones included normative drinking patterns to analyze their influence on alcohol misuse among children in general. Results revealed that parental drinking habits affect early-onset drinking in children [40] and increase the risk of adolescent alcohol misuse and intoxication [41, 42]. Domestically, higher scores on the Children of Alcoholics Screening Test in high school and college students [43] reflect frequent drinking among parents.

Parental alcohol use increases the intensity of alcohol consumption in late adolescent life [44, 45]. Whereas heavy (and moderate frequency) drinking predicts larger amounts of alcohol consumption throughout adolescence [46], the frequency promotes the development of a risky trajectory in adolescent alcohol use [47]. One study reports the predictive effects of paternal drinking frequency and volume on binge drinking among younger adolescents [48]. Some studies identify research gaps in examining associations between parental drinking and child drinking [39, 42]. Research has mostly focused on addressing the association between parental drinking and habitual (chronic risk) adolescent drinking, but little is known about its association with adolescent binge drinking (an acute risk).

Similarly, the effects of parental drinking according to gender differences remain unclear. The study that revealed that paternal drinking frequency and intensity predicted excessive adolescent drinking for younger adolescents, also conceded that higher overall alcohol consumption rates may have influenced the results reflecting the father’s dominant influence [48]. In contrast, many studies have reported the dominant effects of maternal drinking on adolescent alcohol misuse (although fewer reports exist on child binge drinking resulting from the mother’s drinking). For instance, only maternal drinking in two-parent families was significantly associated with adolescent drinking [49], with adolescents drinking more often when maternal drinking exceeded paternal drinking [47, 50].

Women are more susceptible to alcohol-related problems than men, their blood alcohol concentration tends to increase rapidly, and apart from potentially causing fetal alcohol syndrome (from drinking during pregnancy) [51], their problematic drinking behaviors may negatively influence their parenting. That is because mothers (typically) provide much of the child care [52] and better overall supervision, establishing stronger affective and interpersonal bonds with their children [53] compared to fathers, thus, those with drinking habits may exhibit an approving attitude toward their child’s alcohol use [54] and their alcohol consumption recommendations (to the child) may be a risk factor [55]. Therefore, we predict that maternal drinking may influence binge drinking among adolescents.

Another concern related to parental drinking, is how both parents’ drinking influences the risk of binge drinking among adolescents. Previous studies have found that both the mother’s and father’s drinking increased the risk of adolescent drinking [56], and both parents using alcohol, significantly predicted adolescent alcohol misuse [57, 58]. In particular, heavy drinking by both parents predicted an earlier onset and a marked increase in adolescent drinking [46]. However, the influence of both parents’ drinking habits on adolescent binge drinking is unrevealed, but considering existing evidence, the social learning theory may be applicable [59]. Adolescents model their parents’ behavior [60], conceptualizing the process of parental drinking, which encourages adolescent drinking [47]. A Japanese study showed that drinking in front of children had a greater influence than parents’ daily alcohol consumption. Nearly 80% of fathers and over 50% of the mothers in the surveyed sample drank in front of their children [55]. The phenomenon of both parents in a family drinking may be associated with a more favorable attitude toward alcohol use (a longitudinal predictor of alcohol initiation and misuse later) [41], increased availability of alcoholic beverages, and the likelihood of drinking. In fact, most Japanese high school students who drink, find alcoholic beverages at home [61]. Furthermore, parental permissiveness promotes higher binge drinking prevalence [62]. Similarly, research implies that children of parents whose drinking places them in the middle consumption tier, are more exposed to negative outcomes. These include less attention and time to complete homework and irregular bedtime owing to their parent’s drinking [63]. We predict that households where both parents drink heavily, may be more influential in adolescent binge drinking than where only one parent drinks.

Another challenge is whether parental composition (for example, father’s absence and mother’s absence) will affect child binge drinking. Most studies that examine the relationship between parental factors and adolescent drinking fail to separate the father’s and mother’s influence [39], report across parents, or lack information about the father’s drinking [56]. Literature shows that adolescents from two-parent households are less prone to excessive alcohol use (including binging) than those from single-parent households [64], who are more likely to drink [40]. Studies show that adolescents from non-intact families tend to drink more [33], disrupted families are related to child binge drinking [31], and adolescents engage in more frequent heavy drinking (including binging) when they do not live with both their biological parents [32].

Regarding the effects of gender differences (in single-parent households) on children’s drinking behaviors, alcohol use and delinquent behaviors among adolescents were higher in single-father homes than in single-mother homes, owing to lower levels of parental supervision observed in single-father homes [65]. Research shows that children from single (mothers or fathers) parent homes were at higher risk of various behavioral problems (including alcohol use) than children from two-parent families. However, following control for confounders, only the single-father group’s results remained significant [66]. Despite the latter and research showing that living with a single mother was associated with less heavy drinking than a single father [32], one study showed that poor mother-child communication in single-parent families increased hazardous drinking and drunkenness in adolescents [67]. In this regard, it appears that parents’ control and support functions may differ according to gender. A Chinese survey showed: children in non-intact families reported their fathers exerted more paternal behavioral control (knowledge about their child, expectations, supervision, discipline, and demandingness) and their mothers more maternal psychological control (engaging their children’s feelings and thoughts) [68]. Moderate amounts of family support and control would be effective for the socialization and development of sensible drinking in an individual [33], but parents lack in their parenting (including support and control) when drinking excessively [45], potentially causing deviant or excessive drinking behavior in adolescents.

Thus, adolescents from non-intact families are ostensibly associated with binge drinking. Also, since men and women have important but different roles affecting a child’s development, both living without a mother or a father, may hold binge drinking risks for adolescents. Next, we predict that for adolescents not living with either parent influences the prevalence of binge drinking. Related to this, past studies found that children living without both their biological parents engage more frequently in heavy drinking [32, 33, 69]. Importantly, many adolescents do not live with either parent owing to divorce or the death of a parent [70] which may affect their socialization process, since parents are the primary socialization agents during childhood and early adolescence and parents’ values and norms represent the first model for children. Furthermore, limited control and support in deficient families may cause deviant behavior in children [33]. In particular, fragmented families increase the risk of frequent drinking and drunkenness among adolescents [31, 67], while adolescents without parents may have less protective (including parental control) support [48], monitoring, and parental warmth, which could have lowered the risk of binge drinking [34]. Thus, we predict that adolescents who live with neither parent have experienced poor family functions, potentially causing delinquency and risky child behavior, including binge drinking.

Considering the above, we aimed to investigate the relationship between current parental drinking, according to parental composition, and binge drinking among high school students in Japan. Our hypothesis is that parental drinking (mother drinking and both parents drinking) and parental composition (father’s absence, mother’s absence, and neither parent present) would affect adolescent binge drinking. Particularly, we test whether the effects of father drinking and mother drinking separately, as well as the effects of father’s absence and mother’s absence separately, are associated with adolescent binge drinking behavior. Next, we test whether the combined effects of the (a) father and mother drinking and/or (b) father’s and mother’s absence, further influence adolescent binge drinking.

## Methods

### Aim and design

The aim of the Nationwide High School Student Survey on Drug Use and Lifestyle 2018 was to examine the current situation of drug use, including alcohol and tobacco, among Japanese high school students, whose findings informed a drug abuse prevention strategy in Japan. The survey was a cross-sectional study conducted between October 2018 and March 2019.

This study used secondary data from the Nationwide High School Student Survey on Drug Use and Lifestyle 2018 which was conducted by the National Institute of Mental Health in Japan.

### Participants, process, and setting

Participants were high school students randomly selected using a stratified single-stage cluster method, with six regions as the strata and schools as the cluster. Schools were selected using random sampling with probability proportionate to the number of high school students in each region. Self-administered questionnaires were distributed to students in each classroom; these were selected by the person in charge of substance abuse prevention at each school. The teacher in each classroom collected the completed questionnaires and returned them to our research center. A total of 116,313 students at 140 high schools nationwide were surveyed.

### Materials

A self-developed questionnaire (43 items) was used in the original survey. The analysis included demographic and lifestyle variables, information on students’ binge drinking experiences, and their parents’ drinking status and absence, as below.

#### Dependent variable: adolescents’ binge drinking during the previous month

We defined binge drinking as five or more alcoholic drinks for male participants or four or more alcoholic drinks for female participants on the same occasion within two hours [71]. Students were asked, “In the past month, how many days were you at a drinking function (e.g., a gathering that lasted two hours or more) where you had many drinks (five or more drinks for males, four or more drinks for females)?” The response options were: not even once (1), 1 to 2 days (2), 3 to 5 days (3), 6 to 9 days (4), 10 to 19 days (5), 20 to 29 days (6), and every day (7). These responses were recoded as “past month binge drinking,” with response options: no (0) or yes (1).

#### Independent variable: parental drinking according to parental composition

Students were asked the status of their parents’ alcohol use. First, they were asked: “Does your father consume alcohol regularly?” with response options No (1), Sometimes (2), Every day (3), and No father at home (including working away from home, deceased, separated, or divorced) (4). Second, the following question was asked: “Does your mother consume alcohol regularly?” with response options No (1), Sometimes (2), Every day (3), and No mother at home (including working away from home, deceased, separated, or divorced) (4). For both questions, single answers were allowed. If respondents answered, “No father at home” or “No mother at home,” they were not allowed to answer the question related to their parents’ drinking status.

Then, the responses were recoded as a) “Father’s alcohol use status and absence” with response options: FN = Father does not drink (1), FD = Father drinks (2), or NF = No father at home (3), and b) “Mother’s alcohol use status and absence” with options: MN = Mother does not drink (1), MD = Mother drinks (2), or NM = No mother at home (3). We finally combined and re-encoded the two items as “Parental drinking according to parental composition” with response options: FN/MN = Neither father nor mother drinks (1), FN/MD = Father does not drink but mother drinks (2), FN/NM = Father does not drink and no mother at home (3), FD/MN = Father drinks but mother does not drink (4), FD/MD = Both father and mother drink (5), FD/NM = Father drinks and no mother at home (6), NF/MN = No father at home and mother does not drink (7), NF/MD = No father at home and mother drinks (8), and NF/NM = Neither father nor mother at home (9).

The family options were: “both father and mother (two-parent) family” (FN/MN [1], FN/MD [2], FD/MN [4], or FD/MD [5]), father-only family (FN/NM [3] or FD/NM [6]), mother-only family (NF/MN [7] or NF/MD [8]), and neither-parent family (NF/NM [9]).

#### Covariates

Models 2 and 3 included demographic variables that had been associated with adolescent binge drinking in prior research. Covariates included sex, grade [8], lifestyle habits (including getting up and going to the bed at the same time daily, breakfast eating) [63], school life (including enjoying school) [72], family factors (including eating dinner with one’s family, hours spent in the absence of adults, talking with one’s parent about problems) [55, 73, 74], peer factors (including having close friends to hang out with or consult, drinking with peers) [36, 37], thoughts on underage drinking [75], and confidence in refusing a substance when offered [31].

### Statistical analysis

We performed descriptive analysis (using frequency and percentage values) to characterize the sample (Table 1) and assess the distribution of fathers’ drinking and absence status (FN, FD, NF) and mothers’ drinking and absence status (MN, MD, NM) by adolescent binge drinking (Table 2), and the distribution of parental drinking according to parental composition by adolescent binge drinking (Table 3).

**Table 1:**
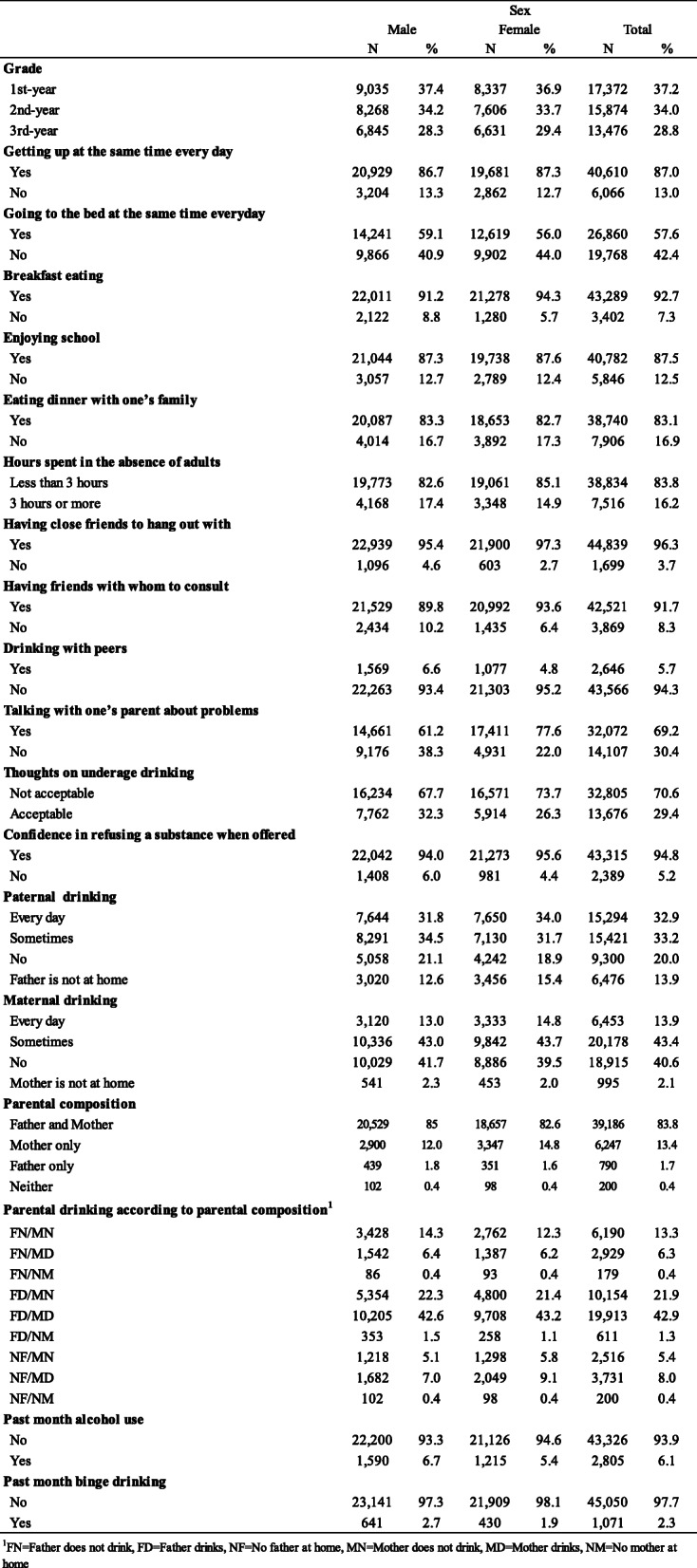
Distribution of respondents according to demographic variables

**Table 2:**
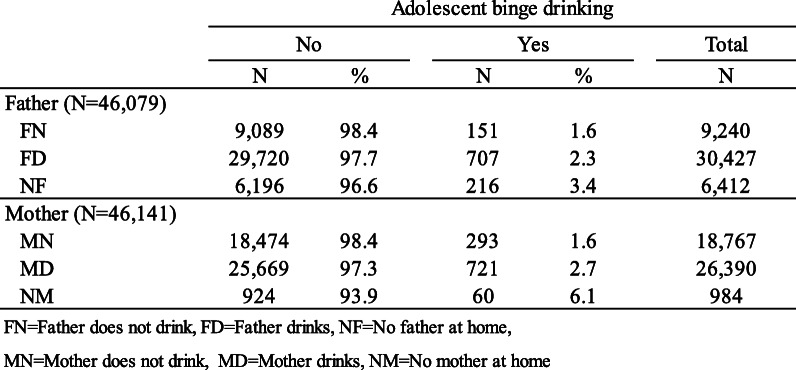
Distribution of respondents according to parental drinking and parental absence by adolescent binge drinking

**Table 3:**
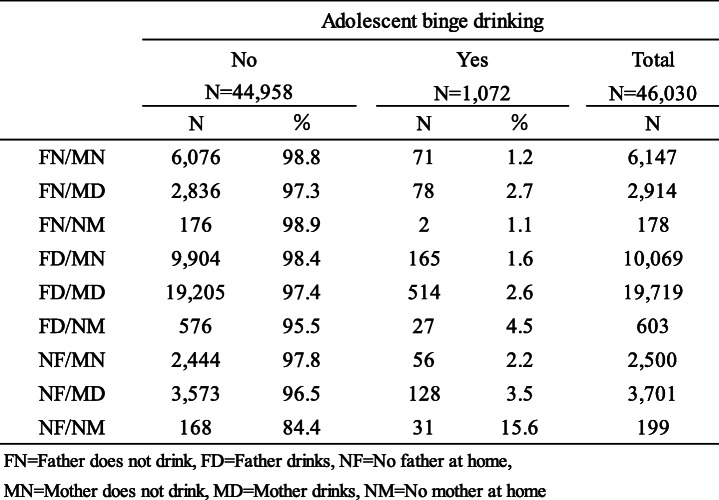
Distribution of respondents for parental drinking according to parental composition by adolescent binge drinking

Subsequently, the association between parental drinking (according to parental composition) and adolescent binge drinking was evaluated in generalized linear mixed models, addressing the clustering of adolescents in sample areas and schools [76]. Multivariable analysis was performed using logistic regression models to test the association. Three logistic regression models were established from unadjusted odds ratios and AORs together with 95% confidence interval (Cl) for the factors. Model 1 included only FD, NF, MD, and NM. Model 2 included variables from Model 1, having been adjusted for all covariates. Model 3 included all variables from Model 1, the interaction between FD and MD, and the interaction between NF and NM combined, after having been adjusted for all covariates.

All data were coded and analyzed using IBM SPSS version 24 (IBM Corp., Armonk, NY, USA). A two-tailed *p* value < .05 was considered statistically significant.

## Results

### Descriptive statistics

The completed surveys (*N* = 47,280; response rate: 56%) were collected from students in 78 schools and 46,848 valid surveys were included for analysis. The demographic distribution of participants (male = 51.6%; female = 48.4%), given in Table 1, shows 37, 34, and 29% are 1st, 2nd and 3rd-year students, respectively. The overall binge drinking prevalence was 2.3%, (male = 2.7%; female = 1.9%) in the preceding month. The prevalence according to the frequency of paternal drinking was 33.2 and 32.9% for occasional and daily drinkers, respectively, but regarding maternal drinking frequency, it was 43.4 and 40.6% for occasional drinkers and “no drinking,” respectively, which means, fathers tended to drink more frequently than mothers. Regarding parental composition, 83.8, 13.4, 1.7, and 0.4% of the respondents live with both father and mother, only their mother, only their father, and neither parent, respectively. The rate of parental drinking according to parental composition showed that nearly half of the respondents (42.9%) were in the FD/MD group, 21.9% in the FD/MN group, and 13.3% in the FN/MN group.

Table 2 shows the distribution of respondents according to parental drinking and absence by adolescent binge drinking. Considering their fathers’ status on alcohol use and absence, 3.4, 2.3, and 1.6% of the respondents from the NF, FD, and FN groups, respectively, were highly experienced at binge drinking. Regarding their mothers’ status on alcohol use and absence, 6.1, 2.7, and 1.6% of the respondents in the NM, MD, and MN groups, respectively, had the most binge drinking experience.

Table 3 shows the distribution of respondents for parental drinking according to parental composition by adolescent binge drinking. Here, 15.6% of the respondents from the NF/NM group, 4.5% of the FD/NM group, 3.5% of the NF/MD group, 2.7% of the FN/MD group, and 2.6% of the FD/MD group, had extensive binge drinking experience.

### Associations between parental drinking according to parental composition and adolescent binge drinking

Table 4 shows the results of logistic regression models reflecting the association between parental drinking according to parental composition and adolescent binge drinking. The results of Model 1 show that the NF, MD, and NM groups were significantly associated with increased likelihoods of binge drinking by adolescents. The results of Model 2 show that only the mother’s status of alcohol use (including MD and NM groups) was significantly associated with the outcome, after having been adjusted for 14 covariates. In Model 3, we examined the FD, NF, MD, and NM groups, as well as the interaction between FD and MD, and the interaction between NF and NM groups, after adjusting for 14 covariates. In the final model, the MD group (AOR: 1.50, 95% CI: 1.06–2.12) and the interaction between NF and NM groups (AOR: 4.35, 95% CI: 2.10–9.02) showed significant associations with adolescent binge drinking. However, other associations between adolescent binge and parental drinking according to parental composition, including the FD group (AOR: 1.25, 95% CI: 0.96–1.63), NF group (AOR: 1.18, 95% CI: 0.84–1.65), NM group (AOR: 1.52, 95% CI: 0.91–2.52), and FD/MD group (AOR: 0.98, 95% CI: 0.61–1.56), were not confirmed.

**Table 4:**
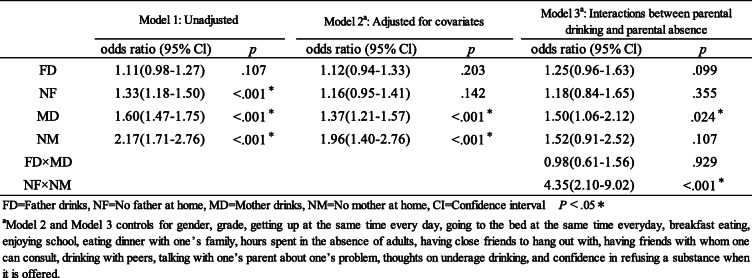
Logistic regression examining associations between parental drinking according to parental composition and adolescent binge drinking

## Discussion

This is the first study to examine the relationship between parental drinking (according to parental composition) and binge drinking in a randomly selected nationally representative sample of high school students in Japan. In adjusted models (including 14 variables) the findings partly supported our hypothesis that adolescents whose mothers drink are more likely to engage in binge drinking. Also, adolescents who were not living with either parent may have been at greater risk of binge drinking.

### Effect of parental drinking

This study revealed that mothers’ drinking habits are significantly associated with binge drinking in adolescents, supporting our hypothesis that maternal drinking is associated with adolescent risky (including binge) drinking. Many studies reported that maternal drinking affect adolescent drinking behavior [47, 49, 50], potentially causing poor maternal parenting (related to support and supervision), promoting a favorable attitude to alcohol, and excessive and frequent adolescent drinking, in particular, when it is problematic [52–55].

Moreover, we mentioned that women are more likely to become problematic drinkers than men [51]. The number of Japanese men with drinking habits have decreased over the past decades, and the gap between their drinking habits and those of women is narrowing [77], possibly because of an increasing number of women with alcohol-related problems. The aforementioned evidence and possibilities may support the theory that mothers’ drinking habits affected adolescents’ risky drinking behavior in this study, just as maternal drinking affects adolescent alcohol misuse. Thus, our results imply that mothers’ drinking may have a greater influence on adolescent alcohol misuse and risky (including binge) drinking, considering alcohol problems specific to women and their consequent reduced role at home.

In our study, fathers’ drinking did not predict adolescent binge drinking. However, previous studies showed frequent and intense paternal drinking predicted excessive adolescent drinking [48], implying that drinking (which is similar to binge drinking) may affect children’s binge drinking behavior. However, normal paternal drinking does not have much effect on children’s risky drinking. In this regard, in Japanese families it is common for fathers to drink, thus implying that drinking is socially acceptable.

Another thought is that drinking habits are very common among fathers; the prevalence of drinking habits among men is overwhelmingly higher than among women [78]. However, the annual decrease in the proportion of male habitual drinkers in Japan is likely to be related to fewer overall problematic drinkers [77], which may be associated with our results. However, in this study, we did not examine the extent or frequency of parental drinking, which prevented us from screening and identifying them as normative or problematic drinkers. Hence, further study is needed.

Our study did not show that living in households where both parents drink may affect binge drinking in adolescents, thus failing to support the related hypothesis. Thus, the findings did not add to existing evidence that both parents (a) using alcohol, significantly predicted adolescent alcohol misuse (increasing the risk of drinking) [56–58], and (b) drinking heavily, predicted earlier onset and a stronger increase in adolescent drinking [46].

We applied the social learning theory to the effects of both parents drinking alcohol on their children’s drinking behavior [47, 59, 60]. Both parents drinking, potentially increases the availability of alcohol at home, promoting a more acceptable attitude toward alcohol use, which potentially stimulates the onset of adolescent drinking in quantity and frequency.

Previous research suggested that there is a very strong association between adolescent drinking and excessive and frequent drinking by both parents [46], and higher levels of parental drinking is associated with increased alcohol availability at home [44]. Thus, the more frequently and heavily parents drink the higher the risk of adolescent binge drinking.

Apart from parental drinking, parenting itself is important since parental control (including monitoring), support, and warmth are crucial against risky drinking behavior [34]. Moderate control encourages children to make decisions and regulate their own behavior, whilst increased warmth and understanding support their autonomy [33]. Parental warmth also enhances bonding with children, potentially providing strong protection against deviant behavior [57]. Thus, even if both parents have drinking habits, well-balanced control, and support from them will enable sufficient socializing of their children to develop their autonomy to confront risky behaviors, including binge drinking.

### Effect of parental absence

The current study showed that paternal absence and maternal absence were not significantly associated with adolescent binge drinking. This result did not support our hypothesis that paternal absence and maternal absence are associated with adolescent binge drinking. Our findings do not support the evidence that non-intact families are more likely to drink [33, 40], disrupted families are related to child binge drinking [31], children of single fathers were at higher risk of alcohol use [66], and that living with a single mother was associated with less heavy drinking than a single father [32]. Alcohol use and delinquent behaviors among adolescents were higher in single-father homes, than in single-mother homes [65]. However, the influence of single-parent households alone does not affect children’s drinking. As mentioned earlier, both parents have important child nurturing roles, requiring parental control and support (including monitoring and warmth), which are crucial to preventing risky drinking behaviors [34]. Growing up in a supportive environment with moderate control, lowers the possibility of risky alcohol behaviors in children. There are several potential explanations why our results did not support existing research. The most likely reason is limited details about the absence of parents, since the definition of “having no parents” includes households where children are living alone owing to work. Divorce and bereavement have negative consequences on children. However, that situation differs from one where parents are separated from their children owing to work. Furthermore, we also did not examine family structures in our study, which may have impacted the result. Other family members or relatives may play the role of the absent parent providing family support and control [33, 45]. A single-parent household survey in Japan, showed that nearly half of single-parent households have cohabitants [79]. Thus, even in households without one parent, other family members could play an important parenting role. Furthermore, divorce rates and family composition differ between cultures. Therefore, it is desirable to discuss parental and extended or immediate family composition to shed light on the reasons for having no parents. Despite these limitations we concluded that the absence of one parent did not affect children’s binge drinking.

Our results revealed that adolescents without either parent are associated with binge drinking, supporting previous findings that children who do not live with both their biological parents consume alcohol more frequently and heavily [32, 33, 69]. In this regard, research indicated that “*custodial grandchildren typically receive care from grandparents because of predicaments among their parents, such as substance abuse, child abuse and neglect, teenage pregnancy, death, illness, divorce, incarceration, and HIV/AIDS*.” [80] Still, we infer that children not living with either parent, were living in a difficult environment compared to at least a single-parent household. Not living with either parent can affect the socialization and development of an adolescent [33] and living in a fragmented family increases the risk of frequent drinking and drunkenness among adolescents [67]. Binge drinking does not appear in families where protective parental (including monitoring, warmth, and parent-child communication) factors are present [34]. Thus, our finding suggested that children who do not live with either parent may be a high-risk group for binge drinking.

### Limitations

There are several limitations to our research. First, owing to its cross-sectional design, we were unable to examine the temporal relationship between parental and child binge drinking; longitudinal design studies are recommended. Owing to secondary data analysis, we were unable to consider several potentially important factors including genetic predispositions, adverse childhood experiences, social influences, and parental mental health. In addition, data regarding parental drinking were collected from the children and depended on their perceptions; therefore, the representativeness thereof may not be accurate. No survey questions regarding the amount and frequency of drinking by parents were included, so we were unable to distinguish whether parents were problematic drinkers or not. In future research, data should be collected from both the parents and children, including items querying the amount and frequency of drinking. Furthermore, our questionnaire did not include items regarding the reasons for single- or neither parent household, therefore the definitions of “single” and “neither” parent were ambiguous. The cause of family deficiency varies depending on country and culture, and hence, which parent group most likely influences Japanese children’s risk behavior needs to be determined. Finally, we did not examine whether parental drinking influenced adolescents according to sex and further longitudinal studies assessing the deferential effects are needed, as past results have been inconsistent [56]. Further research should investigate adolescent binge drinking according to sex differences, clarifying the relationship between drinking in parents and their children. Despite these limitations, this was the first nationwide study to examine the relationship between parents’ drinking (according to parental composition) and adolescent binge drinking. Our findings, that maternal drinking behavior and non-parental families influence binge drinking behavior in Japanese high school students, provide useful information on the parental factors that contribute to binge drinking in these students. We recommend an enhanced prevention program, engaging parents and non-parental family, educating them about the effects of alcohol use on children and proactive family practice. Finally, since this survey was a secondary analysis of the Nationwide Survey, we could not examine all possible factors that influence binge drinking, nevertheless we were able to estimate possible associations among specific variables. We hope that our findings will contribute toward future binge drinking research.

## Conclusions

Using a nationally representative sample of Japanese high school students, the findings of the present research suggest that adolescents whose mothers drink or do not live with them at home may be more at risk of binge drinking. Our findings add substantial evidence to that of previous studies regarding parental drinking and adolescent alcohol misuse. Focusing on parental engagement (including non-parental family members) in future programs and interventions is important in preventing adolescent binge drinking. We hope our findings will prove useful to future binge drinking research. Future analyses should explore whether parental drinking (according to parental composition), considering the amount and frequency of drinking, affects adolescent binge drinking by gender differences.

## Data Availability

The raw data and datasets used and/or analyzed for the current study are not publicly available but are available from the corresponding author on reasonable request. A report on the Nationwide High School Survey on Drug Use and Lifestyle 2018 is also available from the Department of Drug Dependence Research, National Institute of Mental Health, National Center of Neurology and Psychiatry: https://www.ncnp.go.jp/nimh/yakubutsu/index_eng.html.

## Abbreviations

AOR: Adjusted odds ratio
CI: Confidence interval
FN: Father does not drink
FD: Father drinks
NF: No father at home
MN: Mother does not drink
MD: Mother drinks
NM: No mother at home
